# Growth Factors, and Cytokines; Understanding the Role of Tyrosine Phosphatase SHP2 in Gametogenesis and Early Embryo Development

**DOI:** 10.3390/cells9081798

**Published:** 2020-07-29

**Authors:** Muhammad Idrees, Seon-Hwa Oh, Tahir Muhammad, Marwa El-Sheikh, Seok-Hwan Song, Kyeong-Lim Lee, Il-Keun Kong

**Affiliations:** 1 Division of Applied Life Science (BK21 Plus), Gyeongsang National University, Jinju 52828, Korea; idrees1600@gnu.ac.kr (M.I.); ghktjsdh@gmail.com (S.-H.O.); marwa.elsheikh@gnu.ac.kr (M.E.-S.); 2 Institute of Medical Science, University of Toronto, Toronto, ON M5S 1A8, Canada; t.muhammad@mail.utoronto.ca; 3 Department of Microbial Biotechnology, Genetic Engineering and Biotechnology Division, National Research Centre, Dokki, Cairo 12622, Egypt; 4 The King Kong Ltd., Gyeongsang National University, Jinju 52828, Korea; siwd2002@gmail.com (S.-H.S.); 0920-0728@hanmail.net (K.-L.L.); 5 Institute of Agriculture and Life Science, Gyeongsang National University, Jinju 52828, Gyeongnam Province, Korea

**Keywords:** SHP2, growth factors, cytokines, gametogenesis, embryo development

## Abstract

Growth factors and cytokines have vital roles in germ cell development, gamete maturation, and early embryo development. Cell surface receptors are present for growth factors and cytokines to integrate with and trigger protein signaling in the germ and embryo intracellular milieu. Src-homology-2-containing phosphotyrosine phosphatase (SHP2) is a ubiquitously expressed, multifunctional protein that plays a central role in the signaling pathways involved in growth factor receptors, cytokine receptors, integrins, and G protein-coupled receptors. Over recent decades, researchers have recapitulated the protein signaling networks that influence gamete progenitor specification as well as gamete differentiation and maturation. SHP2 plays an indispensable role in cellular growth, survival, proliferation, differentiation, and migration, as well as the basic events in gametogenesis and early embryo development. SHP2, a classic cytosolic protein and a key regulator of signal transduction, displays unconventional nuclear expression in the genital organs. Several observations provided shreds of evidence that this behavior is essential for fertility. The growth factor and cytokine-dependent roles of SHP2 and its nuclear/cytoplasmic presence during gamete maturation, early embryonic development and embryo implantation are fascinating and complex subjects. This review is intended to summarize the previous and recent knowledge about the SHP2 functions in gametogenesis and early embryo development.

## 1. Introduction

The molecular mechanisms in developing multicellular organisms are a captivating phenomenon, inspiring researchers to clarify the ligand-dependent protein signaling pathways that underlie germ cell development, gamete maturation, and the formation of preimplantation embryos as a self-contained system [1]. Over recent decades, our understanding of protein signaling has matured, and several ligand-dependent protein pathways have been identified as playing an essential role during developmental stages. Germ cells, which are the origin of multicellular organisms, arise early in development during gastrulation from the proximal region of epiblast and are recognized as the earliest precursor of gametes [2,3]. Several in vitro studies have reported germ cell reconstitution from embryonic stem cells (ESCs) as well as induced pluripotent stem cells (iPSCs) [4,5]. Hayashi et al. developed primordial germ cell-like cells (PGCLCs), differentiated them to obtain mature gametes, and succeeded in obtaining live healthy offspring [6,7]. The in vivo research or in vitro protocols that were used elucidated that some growth factors and cytokines were required at specific stages for the activation of protein signaling essential for the specification, migration, and proliferation of primordial germ cells, and the further development to mature gametes, capable of fertilization [8,9,10]. 

Several lines of study have identified that these growth factors and cytokines directly bind to their specific receptors present on the surface of the primordial germ cells (PGCs) and activate the signaling pathways essential for cell survival, proliferation, and self-renewal [11,12,13,14,15]. Similarly, the maturation of gametes, an important stage for successful fertilization, also requires several growth factors and cytokines [16,17]. In addition, a plethora of literature is available on the critical role of growth factors and cytokines in fertilization and early embryonic development [18,19,20]. Several studies identified that the preimplantation embryos also require growth factors and cytokines for successful implantation [21,22]. 

SHP2 (previously called SH-PTP2, PTP2C, PTP1D, and SH-PTP3) is a non-receptor protein tyrosine phosphatase that is encoded by protein tyrosine phosphatase non-receptor type 11 gene (PTPN11). A core component of receptor tyrosine kinases (RTKs), cytokines, and G protein-coupled receptor signal transduction, SHP2 shows ubiquitous expression and plays critical roles in cellular growth, survival, proliferation, and migration [23]. In most cases, if not all, SHP2 is required for the activation of mitogen-activated protein kinases/extracellular signal-regulated kinases (MAPK-ERK) and phosphoinositide 3-kinase/protein kinase B (PI3K/AKT) molecular processes and plays a dynamic role in gametogenesis, germ maturation, and embryo development [24,25]. Recent advances in germ cell biology revealed several growth factors and cytokine-dependent regulatory mechanisms; however, the role of SHP2, a major participator of this signaling, has been explored little. Herein, we will focus on the growth factors and cytokine-dependent mechanisms of SHP2 for PGC development, gamete maturation, embryo development, and the nuclear-localized SHP2 role in embryo implantation.

## 2. Literature Review Procedure

### 2.1. Literature Review and Search Strategy 

We extensively searched and studied the growth factors and cytokine-dependent mechanisms of SHP2 in gametogenesis, gamete maturation, early embryo development, and embryo implantation. The selected papers were from different independent databases, including PubMed, Google Scholar, and Web of Science. We used keywords, including “SHP2 AND mechanism”, “SHP2 NEAR Growth factors/cytokines”, “Growth factors/cytokines”, “SHP2 AND/OR Gametogenesis”, “Growth factors, cytokines, early embryo development”, and “growth factors, cytokines, SHP2, embryo implantation” for the literature search. 

### 2.2. Inclusion and Exclusion Criteria 

A. Laboratory rodents of any species were included, but preference was given to the studies performed on mice. Bovines and also human studies were also included. 

B. Ligand-induced protein signaling independent of SHP2 was excluded, but the predicted role of SHP2 in the pathway was included. This prediction was based on the role of SHP2 with the same ligand-receptor and same pathway, but different tissue/organs or organisms. 

## 3. SHP2 Dependent Signaling in Multicellular Organism Development

**Figure 1:**
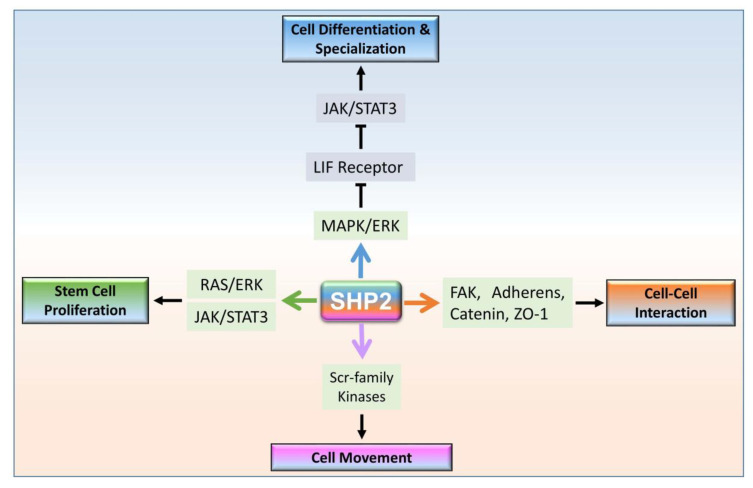
The role of SHP2 dependent signaling in the four essential basic processes of multicellular organism development. SHP2 promotes cell differentiation and specialization, stem cell proliferation, cellular interaction, and movement.

SHP2 plays a prominent role in regulating four basic events of the multicellular organism during embryo development (Figure 1). SHP2 upregulation enhances MAPK/ERK signaling, which suppresses the LIF receptor and as a result downregulate JAK/STAT3 signaling, which ultimately promotes cell differentiation and specialization [26]. Also, SHP2 plays an important role in several types of stem cells’ proliferation by activating RAS/ERK and JAK/STAT signaling simultaneously [27,28]. SHP2 regulates adheren proteins via focal adhesion kinase (FAK) to maintain cell–cell interaction/attachment, specifically in blood–testis gap junctions [28]. Furthermore, tyrosine phosphatase SHP2 increases cell motility through SRC-family kinases. In contrast, SHP2 deficient embryonic stem cells accumulate in the posterior epiblast of the gastrulating embryo rather than migrating through the primitive streak [29,30,31]. Other than development, SHP2 nuclear-cytoplasmic localization is critical for early embryo implantation [32]. SHP2 is mostly expressed in the cytoplasm for signal transduction of numerous receptors, while nuclear-localized SHP2 interacts with transcription factors and regulate their transcriptions. Here, we discuss the cytosolic and nuclear-localized SHP2 mechanisms.

### 3.1. Cytoplasmic Localized SHP2 Mechanisms

Growth factor and cytokine receptors have been found to alter gene expression via interacting with several kinases and phosphatases through their cytoplasmic domains [33,34,35]. Src phosphatases, a major regulatory protein family, play vital roles during the phosphorylation and dephosphorylation states of proteins [36]. SHP2 consists of two Src homology (N-SH2 and C-SH2) domains and one protein tyrosine phosphatase (PTP) domain. Normal cytoplasmic SHP2 is mostly present in two states, the inactive or auto inhibition state, and the active or open state. During the inactive or auto inhibition state, the N-terminal SH2 domain blocks the PTP domain, while in the active state, the SH2 domain binds to specific phosphotyrosine sites of the adaptor proteins of receptors [37]. 

Phosphatases show binding with their physiological substrates, and it was previously identified that the epidermal growth factor receptor (EGFR) is a potent physiological substrate for SHP2 [37]. For phosphatase activity, SHP2 requires tyrosyl phosphorylation (Y542 and Y580) to form a tertiary complex with Gab1/2 (Grb-associated-binding protein 1/2) and the p85 subunit of PI3K, to activate MAP kinases and AKT signaling [31]. In another mechanism, SHP2 directly binds with EGFR and dephosphorylates it at tyrosine 922, which is the binding site for rat sarcoma/GTPase-activated proteins (RAS/GAP) [38]. SHP2 also controls src family kinases (SFK) by regulating their inhibitory tyrosine and the tyrosyl dephosphorylation of multiple SFK substrates, including PLCγ, which leads to activation of the RAS and ERK pathway [39]. SHP2 phosphatase activity is also important for FGF receptor signaling in the activation of MAP kinases [40]. 

Sprouty (Spry), a primary fibroblast growth factor (FGF) signaling core component, is the target for SHP2 to dephosphorylate and detach it from Grb2, and, in this way, the MAP kinase pathway becomes activated [40,41]. In another FGF signaling pathway, the FRS1 linker protein between the FGF receptor and Ras/MAP kinase, forms a complex with Grb2 and SHP2 upon FGFR stimulation. The formation of the SHP2 and Grb2 complex initiates Ras/MAP kinase signaling activation [42]. Insulin receptor substrate 1 and 2 (IRS1 and IRS2) are the central distributors of insulin signaling, and SHP2 was found to be recruited by the insulin receptor for IRS dephosphorylation and bound with PI3K and phospholipase C gamma (PLCγ) for downstream signaling [43].

SHP2 also plays critical roles in cytokine signaling, as this signaling is also essential for the progression of developmental stages [16,44]. Researchers reported that, in response to various cytokines, SHP2 promoted the activation of Janus kinase/signal transducers and the activation of the transcription Janus kinase/signal transducer and activator of transcription (JAK/STAT) pathway [45,46,47]. SHP2 is also known to have a role in the downregulation of the cytokine-mediated JAK-STAT signaling pathway. With regard to the interleukin-6 signaling pathway, SHP2 is recruited by glycoprotein 130, which further leads to regulation of the efficacy and duration of the JAK-STAT signaling pathway [48]. Stem cell factor (SCF), a cytokine, plays an important role in primordial germ cell (PGC) self-renewal, and SHP2 was found to interact with the Kit receptor [49,50]. The binding of SHP2 to c-Kit was expected to be facilitated by a tyrosine residue located in the c-Kit juxta membrane region (Tyr567) [51]. 

SHP2 not only acts on the adaptor protein of receptors for signals transduction but also shows expression and localization in mitochondria. SHP2 plays a significant role in oocyte meiotic maturation by inhibiting mitochondrial degradation via enhancing mTOR dependent mitochondrial biogenesis and inhibiting mitophagy [52]. The inhibition of SHP2 with Phenyl hydrazono pyrazolone sulfonate 1 (PHPS1), which is an active site-directed inhibitor, also significantly reduced meiotic maturation [17]. One study stated that mitochondria localized SHP2 dephosphorylated and inhibited adenine nucleotide translocase 1 (ANT1), which is essential for the nucleotide-binding domain and leucine-rich repeat containing (NLRP3) localization in mitochondria, and this resulted in reactive oxygen species (ROS) induced apoptosis [53].

### 3.2. SHP2 Nuclear Localization and Role in Transcription

Several studies identified nuclear-localized SHP2 and found it to be essential for the proper functioning of those specific tissues. Studies identified that SHP2 nuclear localization is essential for some transcription factors to transcribe their target genes. STAT5a plays an essential role in several genital organs and has a crucial role in early embryo development [54]. Chughtai et al. identified, by nuclear co-immunoprecipitation, that SHP2 makes a complex with STAT5a, and this tight physical and functional interaction is required for the promoter activation of the prolactin receptor signal transduction to the β-casein gene [55]. Nuclear SHP2 also interacts with STAT3, another member of the STAT family, by forming a complex, and negatively regulates its transcriptional activity [56].

SHP2 showed nuclear localization in several reproductive tissues, like our previous study on in vitro oocyte maturation and one study regarding sperm maturation, which stated that SHP2 showed nuclear expression in germ cell-supporting cells (cumulus cells and sertoli cells); however, the function of nuclear-localized SHP2 still requires exploration in those tissues [17,28]. Ran et al. found that nuclear-localized SHP2 in the uterus was essential for the estrogen receptor-α (ER-α) nuclear transcription of progesterone (*pgr*) gene in mice, and for the tissue (uterus)-specific SHP2 knockout to completely inhibit embryo implantation [32]. To find the mechanism of nuclear SHP2 in the uterus, the authors found that SHP2 augmented the Src kinase-facilitated ERα tyrosine phosphorylation, which assisted ERα in binding to its target *pgr* promoter, and, consequently, activated the ERα transcription of progesterone in preimplantation uteri [57]. A complex between SHP2 and ERα was also discovered in one study where they observed that an SHP2 knockdown significantly reduced the ERα transcriptional activity [57]. ERα in the nucleus, and also extra nuclear ERα, formed a complex with SHP2 and mediated MAP kinases and AKT signaling, while an SHP2 knockdown significantly reduced that signaling [57]. 

**Figure 2:**
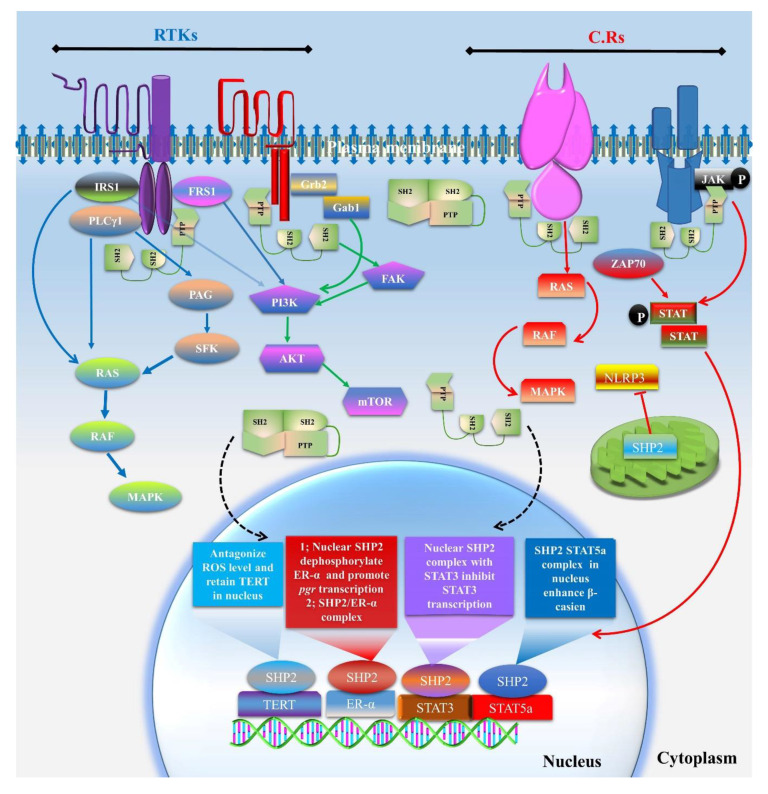
SHP2 dependent growth factors and cytokines receptors (CRs) signal transduction and interaction with nuclear factors. SHP2 is essential for regulating several key ligand-dependent pathways and also participate in the activities of several transcriptional factors. Insulin receptor substrate (IRS) dependent RAS/RAF/MAPK has been identified, and SHP2 was found to play a significant role in this signaling. Phospholipase C gamma (PCLγ) also activates RAS/MAPK via PAG and SFK, which also need SHP2 for this signal transduction. Far1-related sequence (FRS1) has been activated by FGF ligand via FGF receptor, and FRS1 dephosphorylation by SHP2 is a known mechanism for FGF signaling. Growth factor receptor-bound protein 2 (Grb2) and Grb-associated-binding protein (Gab1) were found to be activated by EGF and a few other ligands, and SHP2 shows its association with them for the activation of MAPK and AKT signaling. CRs adaptor proteins like Janus kinase (JAK) and Zeta-chain-associated protein kinase 70 (ZAP70) also need SHP2 for MAPK and STAT signaling. Other than these signaling SHP2, it has also been identified in mitochondria, resisting NLRP3 localization and mitochondrial toxicity. Nuclear localized SHP2 active or auto inhibition state is as yet unknown, and also during complex formation with other transcription factors, but several studies have identified the interaction of SHP2 with TERT, ER-α, STAT3, and STAT5a.

Nuclear SHP2 also demonstrated an association with telomerase reverse transcriptase (TERT) in the nucleus, as H_2_O_2_ treatment exported TERT from the nucleus and enhanced cytotoxicity [58]. SHP2 overexpression in the nucleus enhanced the tyrosine 707 phosphorylation of TERT and inhibited its nuclear export [58]. The involvement of the active or auto inhibitory state of SHP2 in complex formation with nuclear proteins and extra nuclear proteins and also the main residues of interaction are the issues yet to be resolved (Figure 2).

## 4. Growth Factors and Cytokines Dependent Signaling in Primordial Germ Cells (PGCs) and SHP2 Functions

The origination of a new organism starts from germ cells, as these cells are the dynamic source for genetic diversity and evolution. Germ cells are formed during early embryogenesis, shortly after the implantation of the embryo, and they later initiate meiosis to give rise to oocytes and spermatocytes. PGCs originate from the epiblast cells before the epiblast splits into three germ layers (the ectoderm, endoderm, and mesoderm), and then cluster at the base of the incipient allantois in the extraembryonic mesoderm [59]. The mechanism of germ cell lineage begins from bone morphogenic protein (Bmp) via binding to and bringing together type I (activin receptor-like kinase 3/BmprIA) and type II (Bmp type II receptor and activin type II receptors (ActrIIA and ActrIIB)) receptors on the cell surface, as these receptors activate pathways essential for germ cells differentiation from surrounding somatic cells [60]. Stem cell factor/cluster of differentiation 117 (SCF/CD117 or KL/KIT) interaction was also found as an important ligand-dependent pathway for the specification of PGCs from the surrounding somatic cells during embryogenesis [50,61].

### 4.1. Role of Growth Factors and Cytokine in PGCs Specification, Migration and Proliferation

The roles of growth factors and cytokines have been identified by in vitro mimicked studies, in which embryonic stem cells (ESCs) or induced pluripotent stem cells (iPSCs) were used to derive PGCs [7,9]. Even the specification of PGCs from epiblast-like cells or ESCs or iPSCs requires BMPs and bFGF (basic fibroblast growth factor) [62,63]. Other than growth factors, a cytokine presence, like the leukemia inhibitory factor (LIF), is necessary for PGC development [11]. After specification at allantois, PGCs must migrate toward genital ridges and proliferate [64,65]. The process of PGC migration and the formation of gonadal and testicular ridges involves several growth factors and cytokines, as identified by numerous studies [66,67,68,69,70].

FGF2 (bFGF), a very basic growth factor for PGC specification, also plays an essential role in germ cell migration. Research has identified that bFGF acts through FGFR1-IIIc and enhances MAP kinases in migrating germ cells [65]. After their arrival at the genital ridges, PGCs begin to proliferate, which also requires growth factors and cytokines; however, the in vivo mechanism of growth factors and cytokine interactions with PGCs is still unexplored. In vitro mimicked studies revealed the importance of these factors for PGC proliferation [63]. Resnick et al. successfully proliferated mouse PGCs in culture media with the addition of bFGF, SCF, and LIF [71]. The Kit ligand and its receptor, a member of the receptor tyrosine kinases (RTKs), have long been known to be related to the proliferation and survival of PGCs [72,73]. Other studies identified that mast-cell growth factor (MGF) and tumor necrosis factor-alpha (TNF-α) stimulated and proliferated PGCs in culture media [13,15].

### 4.2. SHP2 Expression and Interaction Prediction with Growth Factors and Cytokines Receptors Responsible for PGCs Specification, Migration and Proliferation

The above-mentioned studies revealed the importance of growth factors and cytokines for PGC specification, migration, and proliferation. However, the mechanisms of action for these factors are mostly unidentified, and we have no information regarding the underlying intracellular pathways that link the extracellular signals to the transcriptional regulation in PGC specification, migration, and proliferation. If the effect of these factors is receptors-dependent, then SHP2 must have a central role in signal transduction and would be one of the key factors for the specification, migration, proliferation, and differentiation of PGCs. This extrapolation is because, in several tissues, the interactions between SHP2 and all of these growth factors and cytokines that play roles in PGC specification, migration, and proliferation are already identified, as shown in Table 1 and Table 2 [37,74,75,76]. Thus, this domain of germ cell biology requires experimental exploration to understand the behavior of PGCs.

## 5. Oogenesis and SHP2 Dependent Growth Factors and Cytokines Signaling 

Oogenesis is the process of the differentiation of PGCs into functional female germ cells (oocytes). The post-migratory PGCs, after reaching the gonads, mitotically proliferate and begin meiosis to become oogonia. In mammals, oogenesis consists of (a) the colonization of PGCs in the gonads, (b) the differentiation of oogonia, (c) the proliferation of oogonia, (d) the initiation of meiosis, and (e) the arrest at the diplotene stage of meiotic prophase 1 [91]. During the follicle formation, oogonia are surrounded by a thin layer of gonadal somatic cells and form a compact shape. In the female reproductive system, folliculogenesis is a crucial step and requires specific gene expressions at different developmental stages. A previous investigation analyzed the gene expression dynamics of early ovarian folliculogenesis and found that the insulin growth factor 1 receptor (IGF1R), notch homology 1 translocation-associated (NOTCH), and vascular endothelial growth factor and its receptor (VEGFR) signaling pathways were overexpressed in supporting granulosa cells as well as in oocytes [89]. Other studies found that the deletion of GDF9 inhibited somatic cell formation and differentiation into theca and granulosa cells [90,92]. These growth factors and cytokine dependent pathways were found to be essential for perfect oogenesis.

The in vitro reconstitution of ovaries is currently a highly researched area, and several researchers found that growth factors and cytokines were essential for in vitro oogenesis and for successful oocyte development [83]. Some of the growth factors and cytokines, like the presence of bFGF and LIF in media, were essential for PGC interactions with somatic cells and for the formation of in vitro oocytes. Both factor receptors transduced their signals by interacting with SHP2 [85,93]. Stem cell factor (SCF), another important cytokine for oogenesis, also interacted with SHP2 through the SCF receptor (Kit) for signal transduction [76,94]. However, the SHP2 expression and growth factor- and cytokine-dependent functions of SHP2 still require exploration in oogenesis.

### 5.1. SHP2 Dependent Growth Factors and Cytokines Role in Oocyte Meiotic Resumption, Maturation and Ovulation

In order to obtain oocytes capable of fertilization, female germ cells require multiple processes, such as the activation of the primary follicles via luteinizing hormone (LH) surge, the meiotic maturation of oocytes, and ovulation. All these processes require several specific growth factors and cytokines; however, the mechanisms of these growth factors and the cytokine-dependent initiation of oocyte development are highly unmapped. Here, we give some of the known mechanisms and also linked them with SHP2.

#### 5.1.1. Activation of Primordial Follicle and Role of Growth Factors and Cytokines 

**Figure 3:**
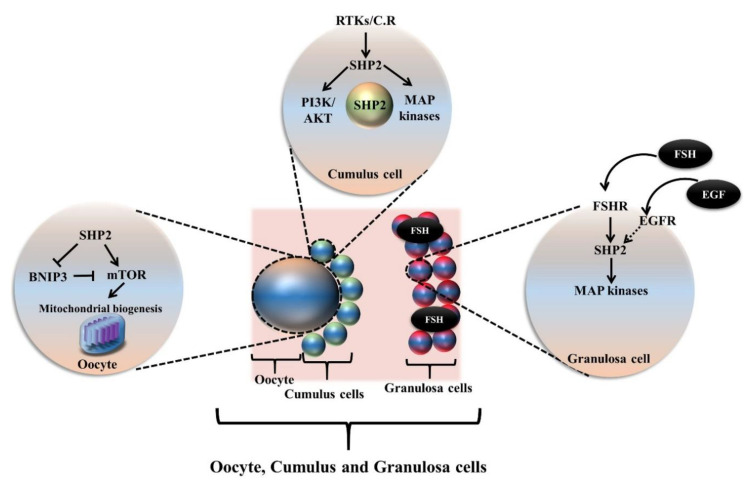
Oocyte maturation and ovulation is a critical process for female fertility. SHP2 is found to be critical for the FSH signaling in granulosa cells and its inhibition significantly reduced MAP kinases. SHP2 also shows expression in cumulus cells, and it has been identified that SHP2 is nuclear localized in the germinal vesicle stage cumulus-oocyte complex. SHP2 expression becomes enhanced by EGF, FGF, and LIF addition to maturation media during oocyte in vitro maturation. Other than that, SHP2 is also involved in negatively regulating the mitophagy, of the oocyte, by enhancing mTOR induced mitochondrial biogenesis and inhibit BNIP3.

In females, the oocytes arrest at the diplotene stage of the first meiotic prophase, at around birth, along with a thin layer of granulosa cells [52]. Granulosa cells undergo highly dynamic changes during folliculogenesis and play a vital role in oocyte maturation. The communication between the oocyte and somatic cells is bi-directional and occurs via gap junctions and paracrine signaling. Follicle stimulating hormone (FSH) and luteinizing hormone (LH) are required for oocyte meiotic resumption and maturation [77]. EGF-like growth factor was found to be a mediator of LH action and to play a significant role in oocyte meiotic resumption [95]. Several studies identified the indispensable role of growth factors and cytokines not only in meiotic resumption but also for in vivo and in vitro oocyte meiotic maturation [18,80,96]. One study found that several receptor tyrosine kinase (RTK) ligands, like the Kit ligand, IGF1, EGF, and PDGF, were involved in triggering the phosphatase and tensin homolog (PTEN)-PI3K-AKT pathway in granulosa cells and activating the primordial follicle [97]. All the above mentioned ligand receptors showed interactions with SHP2 and active research is required to explore the SHP2 dependent mechanisms of these growth factors and cytokines (Figure 3).

#### 5.1.2. SHP2 Dependent Growth Factors and Cytokines Signaling during Oocyte Meiotic Maturation

RTK ligands play an important role in the maturation of oocytes, and the downstream signaling of these receptors, PI3K/AKT and MAPK/ERK, were found to be essential for germinal vesicle breakdown and nuclear maturation [29,31]. A plethora of literature is available on the well-known role of growth factors and cytokines for oocyte meiotic resumption and maturation, but very few studies have explored the mechanisms of action of these factors. SHP2 shows expression in the ovaries of several species; however, up till now, no study completely explored the conventional or unconventional functions of SHP2, by deleting SHP2 from the ovaries or ovary specific tissues [17,98]. One study identified SHP2 expression in granulosa cells and found that its function was essential for FSH signaling [99]. We previously found that EGF significantly enhanced SHP2 expression (cumulus-oocyte complex) during oocyte meiotic maturation [17]. The site-directed inhibitor (PHPS1) of SHP2 significantly reduced oocyte maturation, which demonstrates that active SHP2 is involved in oocyte meiotic maturation. MAP kinases and PI3K/AKT signaling plays a vital role in oocyte maturation, and research has found that active SHP2 inhibition significantly reduced the signaling [17]. One study also identified the SHP2 functional role in oocyte mitochondria and concluded that SHP2 blocked mitochondrial degradation via inhibiting mitophagy [52]. SHP2 is a known upstream of the Akt/mammalian target of rapamycin (mTOR) and ERK signaling pathways in multiple cellular contexts, and several studies identified their crucial roles in oocyte maturation, embryo development, and female fertility [31,100]. SHP2 requires further exploration to understand the oocyte behavior and to improve assisted reproductive technology techniques.

#### 5.1.3. SHP2 Dependent Growth Factors and Cytokines Signaling during Oocyte Ovulation

The molecular mechanisms of ovulation are still far from being completely understood; however, several studies have found that luteinizing hormone (LH) initiates a signaling cascade in which nuclear progestin and its receptor (PGR) activation are the main phenomena that target various downstream signaling pathways leading to follicular rupture [101]. The release of FSH was also found completing the follicular rapture, and SHP2 showed its involvement in FSH signal transduction, but none of these studies are yet available on this issue [99,102]. SHP2 showed expression in granulosa and cumulus cells, interactions with the LH pathways, and a role in oocyte ovulation may be possible [17,99,103]. Several studies also identified that LH and FSH both enhanced EGFR signaling, and this pathway is critical for follicle rupture [102,104]. The role of SHP2 (a core component of EGFR signaling) in follicular rupture requires exploration, and the direct link between LH signaling and SHP2 functioning may be beneficial for hormonal imbalance-induced infertility or polycystic ovary syndrome.

## 6. The Contribution of SHP2 to Spermatogenesis, Spermatogonia Stem Cells (SSCs) Self-Renewal and Differentiation

The process of spermatogenesis results in the formation of the haploid male gametes required for the fertilization of an oocyte. Spermatogenesis on the molecular level is much more explored compared to oogenesis, and several studies have been conducted on the functional role of SHP2 in spermatogenesis. Unlike oogenesis, spermatogenesis is a continuous process that has three major steps; (a) mitosis (spermatocytogenesis) and the proliferation of spermatogonia stem cells (SSCs); (b) meiosis (spermiogenesis), by which the chromosome number is reduced from diploid to haploid; and (c) differentiation of the round spermatid into the spermatozoon. The proliferation and differentiation of PGCs occur in embryonic testes until they enter into mitotic arrest and differentiate into pro-spermatogonia [105,106]. Numerous growth factors and cytokines are required for the differentiation of PGCs into spermatogonia stem cells, embryonic testis development, differentiation of the supporting somatic cells, and formation of testicular cords that enclose these germ cells [16,107]. The spermatogenic capacity of cells of interest, particularly PGCs, can also be assessed through the creation of a reconstituted testis [108,109].

**Figure 4:**
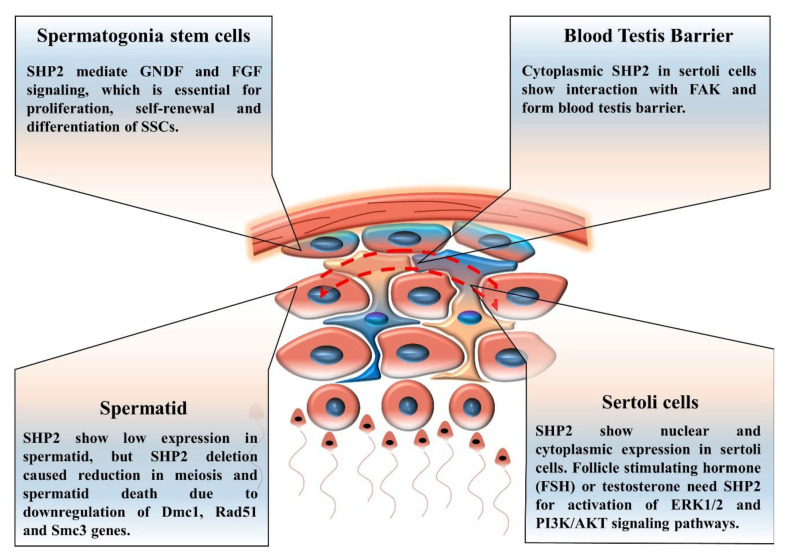
Scheme depicting the generation of mature sperm from spermatogonia stem cells and the key pathways involved. SHP2 expression in sertoli cells, spermatogonia stem cells, and involvement of SHP2 in blood–testis barrier formation. SHP2 also show nuclear localization in sertoli cells, but the function is unknown.

SHP2, a downstream and main regulator of RTK signaling, showed expression in spermatogonia stem cells and in the somatic supporting cells of the testis, as shown in Figure 4 [81]. During the process of spermatogenesis, the generation of haploid spermatozoa differentiated spermatogonia stem cells and SHP2 played a key role during this differentiation [81]. Several growth factors and a cytokine presence were also important for haploid spermatozoa formation; however, growth factors and cytokine-linked SHP2 functions are yet unexplored [16]. SHP2 and growth factors are not only necessary for differentiation but also for self-renewal and the expansion of spermatogonia stem cells [62,110].

Germ cells supporting somatic cells (sertoli cells) produced growth factor-like glial cell-derived neurotrophic factor (GDNF) and bFGF for the proliferation of spermatogonia stem cells and the production of mature sperm [82,111,112]. These sertoli cells (SCs) not only produce growth factors and cytokines, but also express receptors of these ligands to facilitate the differentiation of germ cells to spermatozoa via direct contact and by regulating the environment milieu in seminiferous tubules [113]. SHP2 showed nuclear and cytoplasmic expression in SCs, and its active mutant (Q79R) stimulated the ERK1/2 pathway [28]. The authors concluded that SHP2 is a key regulator of the blood–testis barrier (BTB) formation, by interacting with focal adhesion kinase (FAK), and also that SHP2 played a role in the localization of N-cadherin, β-catenin, and ZO-1 toward the plasma membrane [28]. However, the nuclear-localized SHP2 functions in SCs still remain obscure. Another study demonstrated that SHP2 regulated testosterone, FSH, and EGF signaling in SCs [114]. An SC-specific SHP2 knockout disturbed the blood–testis barrier and reduced the germ cell quantity in mice. The mice also exhibited SC-specific infertility [114].

### Spermatogonia Stem Cells (SSCs)

SSCs are undifferentiated spermatogonia cells, and they have pluripotency as they maintain their own population and also differentiate into spermatozoa [84]. Growth factors and cytokines mediate intracellular signaling in SSCs, and research identified that active SHP2 inhibition reduced the intracellular signaling that regulated SSC proliferation, self-maintenance, and differentiation into mature sperm cells [81]. Previously, researchers identified that an SHP2 knockout from SCs affected the seminiferous tubules and damaged SSCs, by initiating excessive differentiation and deteriorating their ability for self-maintenance [114].

SHP2 stimulates several signaling pathways, like PI3K/AKT, MAP kinases, and JAK/STAT signaling [85]. The JAK/STAT pathway is necessary for male germ cells and also for male genital organ formation [115,116]. In SSCs, the JAK/STAT pathways play essential roles in self-renewal and differentiation, and SHP2 may regulate LIF-induced JAK/STAT signaling in SSCs [81,86].

## 7. Early Embryonic Development and SHP2 Mediated Signaling Network

The origination of multicellular systems, including humans, arises from single-celled totipotent zygotes, which undergo rapid cell division and form a preimplantation embryo. During preimplantation development at the blastocyst stage, the embryo comprises two distinct cell lineages: the outer epithelial cell layer, known as the trophectoderm (TE), and a cluster of cells attached to one side of the inside surface of the TE, known as the inner cell mass (ICM). The remainder of the blastocyst is taken up by the fluid-filled blastocoel cavity [117].

Growth factors and cytokines play significant roles in preimplantation embryo development. Hundreds of in vitro studies identified numerous growth factors and cytokines essential in culture media for successful and healthy embryo development [17,22,87,118]. SHP2 demonstrated expression in preimplantation embryos, and several studies linked its conventional role with growth factors and cytokines [17,23,119]. One study provided experimental proof that SHP2 gene knockout embryos died during preimplantation development, while truncated SHP2 embryos died during implantation [23]. The authors concluded that SHP2 is necessary for preimplantation embryogenesis and, particularly, for trophoblast stem cells (TE) by regulating FGF4 induced SFK/RAS/ERK signaling and inhabiting Bcl-2 like protein 11 (Bim) initiated apoptosis [23].

TE cells are essential for implantation of the embryo by invading the uterine tissue, the formation of trophoblast lineages represented in the placenta, and the exchange of nutrients and waste between the embryo and the mother [120]. It has been identified that the addition of FGF4 differentiated TE cells, while SHP2 inhibition completely blocked the FGF4 signaling pathway [23]. We previously identified that active SHP2 inhibition with PHPS1 (a site-directed inhibitor), during in vitro embryo culture, reduced the total number of cells, enhanced apoptosis, and also inhibited embryo implantation [17]. A SHP2 knockout or inhibition at the embryonic stage not only reduced embryo implantation but also enhanced pro-apoptotic signaling [78,79].

### SHP2 Role and Mechanism in Embryonic Stem Cells (ESCs)

ES cells are pluripotent stem cells with the ability to differentiate into every type of cell [121]. Growth factors and cytokines regulate stem cell properties, and SHP2, a core component of growth factors and cytokine signals, plays a substantial role in ES cell self-renewal and differentiation [86,122]. Several factors, like BMP4, bFGF, and LIF, are critical for the regulation of ES cells pluripotency. SHP2 genetic ablation in mouse ES cells stimulated BMP4-induced SMAD 1/5/8 signaling by increasing the expressions of the inhibitor of differentiation (ID) 1 and 3 as compared to the control. In contrast, human ES cells showed impaired SMAD signaling in SHP2 knockout cells when stimulated with BMP4 [85]. FGF signaling pathways promoted ES cell differentiation, and SHP2 activation by FGFR inhibited sprouty1, resulting in increased FGF/ERK signaling and enhanced ES cell differentiation [123].

Cytokines play a critical role in ES cell self-renewal and differentiation. LIF, a cytokine, stimulates JAK/STAT3 signaling, which is vital for ES cell differentiation and self-renewal, and SHP2 plays a conventional role in LIF mediated JAK/STAT3 signal transduction [86]. In contrast, one study identified SHP2-dependent negative regulation of the LIF/JAK/STAT3 pathway [26]. SHP2 activation enhanced ERK signaling, which repressed LIF receptor expression and resulted in the suppression of LIF stimulation of JAK1/STAT3 signaling. SHP2 inhibition facilitated the maintenance of mouse ES cell self-renewal due to increased STAT3 expression [26]. Another study stated that LIF-associated ES cell self-renewal was not associated with the gp-130 and SHP2 complex-mediated activation of STAT3 [124]. One study concluded that SHP2 dysregulation could be compensated by other signals for ES cell self-renewal [125]. Another study stated that SHP2 knockout ES cells suppressed three germ layer cell lineages and also suppressed the STAT3 and ERK pathways [85].

## 8. Nuclear/Cytoplasmic Localization of SHP2 and Embryo Implantation

In mammals, embryo implantation is one of the most critical steps of reproduction, and implantation failure constitutes a major cause of infertility. A synchronized dialogue is essential between the embryo at the blastocyst stage and a receptive endometrium for successful implantation. Ovarian steroids and progesterone exclusively control the embryo implantation, and growth factors and cytokines were found to play an essential role as mediators of implantation in mammalian species [21]. The involvement of RTK-coupled cytokines and growth factors in the interaction and cross-talk between embryos and maternal tissue has been a hot topic in the last few years. Previously, research has identified that growth factors and cytokines exert their actions by endocrine, paracrine, and juxtacrine mechanisms during implantation [126]. A number of growth factors and cytokines, like EGF family growth factors, LIF, and IL 11, are essential for normal embryo implantation, and the signaling pathways activated due to these factors are important players in directing the normal early pregnancy events [88,127,128,129].

**Figure 5:**
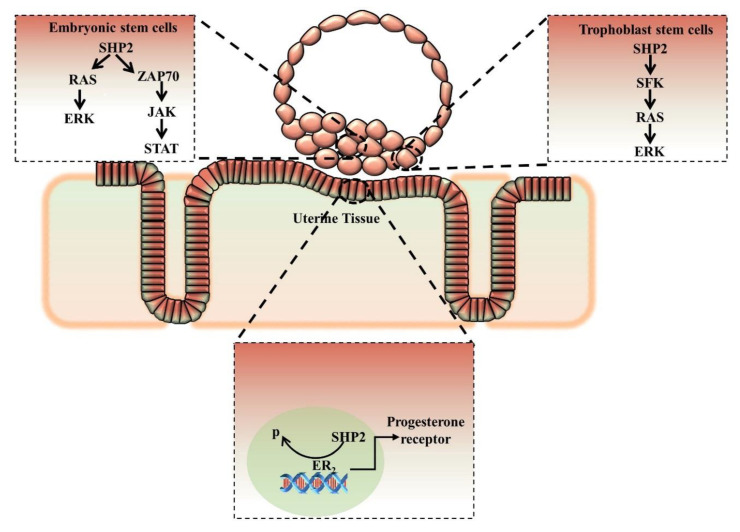
Schematic representation of embryo implantation. SHP2 is expressed in inner mass cells and outer trophoblast cells. SHP2 regulates FGF4 signaling in trophoblast stem cells proliferation and differentiation, and also participates in invasion of the mother uterus. Uterine SHP2 is nuclear-localized and regulates ER-α transcription of progesterone. SHP2 inhibition or knockout either from embryo or uterus completely blocks embryo implantation.

Several studies identified that SHP2 plays an important role in embryo implantation by activating several pathways in the embryo and also in the mother’s uterus (Figure 5). SHP2 expression is critical for trophoblast stem cell proliferation and differentiation, and trophoblast cells are specialized placenta cells with the property of maternal tissue invasion [78]. One study identified the unconventional nuclear expression of SHP2 in mouse uterus and human Ishikawa cells (endometrium). The authors found that uterine specific SHP2 deletion reduced ERα transcription and inhibited the uterine-specific progesterone receptor expression, which is essential for embryo implantation [32]. The authors claimed that nuclear SHP2 activated ERα by enhancing its Tyr phosphorylation, dephosphorylating an inhibitory Tyr in Src kinase, and recruiting ERα to its target gene [32]. SHP2 deletion from either the embryo or from the mother’s uterus completely inhibited implantation. SHP2 has a well-known role in cellular invasion and metastasis in cancer mostly due to gain-of-function mutations; however, during implantation, the role of wild SHP2 in cellular invasion still requires exploration [98,130].

## 9. Conclusions and Future Directions of SHP2 Research

Infertility is a major health care problem, and one out of seven couples trying to have a baby will experience infertility. Several proteins have been identified as playing essential roles in male and female fertility [131,132]. SHP2 is a primary component of growth factors and important in cytokine signal transduction, and this review demonstrated the involvement of SHP2 in the control of various regulatory levels during gametogenesis, embryo development, and implantation. SHP2 and SHP2-related signaling molecules can be used for the characterization, prediction, and regulation of ovarian functions, as well as for the diagnostics and treatment of reproductive disorders. SHP2 germline mutations lead to developmental disorders, and somatic mutations lead to leukemogenesis [133]. Gain-of-function (GOF) mutations or loss-of-function (LOF) mutations in the SHP2 encoding gene PTPN11 affect both male and female fertility. In SHP2, GOF mutations produced Noonan syndrome, involving hypo-spermatogenesis with reduced seminiferous tubules and immature SCs [134]. SHP2 LOF mutations during LEOPARD (lentigines, ECG conduction abnormalities, ocular hypertelorism, pulmonary stenosis, abnormal genitalia, retardations of growth, and deafness) syndrome also induced infertility [135].

SHP2 has been highly explored in oncology and development; however, the relation between SHP2 and fertility, and particularly female fertility, still requires exploration. Numerous upstream and downstream proteins of SHP2 have been identified, and several functions, including activation, auto inhibition, mutation, phosphorylation, localization, and complex formation with several proteins, have also been explored. SHP2 related signaling can be an easy target due to a plethora of computational, structure, and function studies and due to its predicted and explored role in germ cell development, gamete maturation, embryo development, and implantation.

## Figures and Tables

**Table 1:** Growth factors SHP2 dependent signaling and involvement in gametogenesis, gametes maturation, early embryo development, and implantation.

Growth Factors	Signaling Cascade	Signaling Target in Gametogenesis and Early Embryo Development	SHP2 Role in Signaling Identified in Other Tissues	Key References
EGF	EGFR/Grb2/SHP2/p85 PI3K/AKT RAS/MAPK	Play a role in early ovarian folliculogenesis	SHP2 make complex with Grb2 and p85 to activate PI3K/AKT signaling	[17,30,77,78]
		Resume meiosis and mediate FSH signaling in the oocyte. Play a role in embryo implantation	Dephosphorylate EGFR on Tyr 922 to activate RAS/MAPK.	
bFGF	FGFR/ Spry/Grb2 MAPK	bFGF plays a role in PGCs specification, migration, and proliferation	SHP2 dephosphorylate Spry and detach it from Grb2 and activated MAP kinases.	[61,63,66,69,79]
		PGCs proliferation and self-renewal. Enhance in vitro oocyte maturation and embryo development.	FRS1,Grb2 & SHP2 make complex to activate RAS/MAPK	
IGF	IGFR/ IRS/MAP K/ERK	Play a role in early ovarian folliculogenesis	SHP2 dephosphorylate IRS1/2 and recruit its binding with PI3K and PLCγ	[19,40,80]
		Enhance invitro oocyte maturation and embryo development		
GDNF	GDNF RET/PI3K/AKT	Spermatogonia stem cells self-renewal and proliferation	SHP2 interact with RET and activate PI3K/AKT signaling	[60,81,82]

**Table 2:** Cytokine signals and SHP2 activation in gametogenesis and early embryo development and implantation.

Cytokines	Signaling Cascade	Signaling Target in Gametogenesis and Early Embryo Development	SHP2 Role in Signaling Identified in Other Tissues	Key References
BMPs	BMP receptors type I and type2 SMAD4/1/5/8	BMPs induce the formation of PGCs from epiblast of the embryo	Interaction between BMP receptors and SHP2 is yet not completely explored, SHP2 deletion in human ES cells impair SMAD signaling.	[58,60,61,83]
LIF	LIFR Gp-130/zap-70 JAK/STAT	PGCs development, proliferation, and oocyte in vitro maturation	SHP2 interact with gp-130 and also with Zap-70 for the activation of JAK/STAT and MAP kinases	[65,69,72,78,79,83,84]
		Play a role in embryo implantation		
KL/SCF	Kit receptor PI3K/AKT RAS/MAPK PLCγ/Adherine	PGCs proliferation, self-renewal, and oocyte maturation.	SHP2 bind with c-Kit at Tyr567 located in the c-Kit juxta membrane region	[46,47,59,70,71,74,85]
Colony stimulating factor CSF-1	CSF1R MAPK/ERK	Play a role in oocyte meiotic resumption, and maturation	SHP2 become phosphorylated and activated by CSF-1 receptor for ERK pathway activation	[79,86,87]
Interleukin	ILR Gp-130 JAK/STAT	Enhance inner cell mass of embryo during in vitro development	SHP2 bind with gp-130 a receptor sub-unite of interleukins and suppress JAK/STAT pathway via downregulating JAK activity	[22,45,88]
		Play a role in embryo implantation		
TGF-β	TGFR JAK2/STAT3	Gonadal and testicular development	TGF-β activate SHP2 and recruit it to JAK2 for dephosphorylation at Y570, resulting in activation of STAT3	[19,44,64]
		Play a role in early embryo development		
GDF9	BMPR2/ ALKA4/5/7 SMAD2/3/4	Play a role in follicle formation during the early stage, and its deletion inhibit somatic cell differentiation with complete infertility.	Interaction between GDF9 receptor and SHP2 is yet unidentified, but both the proteins show expression in granulosa cells and downstream pathway is MAP kinases	[89,90]
